# The Cambridge Prognostic Groups for improved prediction of disease mortality at diagnosis in primary non-metastatic prostate cancer: a validation study

**DOI:** 10.1186/s12916-018-1019-5

**Published:** 2018-02-28

**Authors:** V. J. Gnanapragasam, O. Bratt, K. Muir, L. S. Lee, H. H. Huang, P. Stattin, A. Lophatananon

**Affiliations:** 1 Academic Urology Group, Department of Surgery and Oncology, University of Cambridge, Box 279 (S4), Cambridge Biomedical Campus, Cambridge, CB2 0QQ UK; 2 Addenbrookes Hospital, Department of Urology, Addenbrooke’s Hospital, Cambridge, UK; 3 Department of Translational Medicine, Division of Urological Cancers, Lund University, Lund, Sweden; 4 Institute of Population Health, University of Manchester, Manchester, UK; 5 Department of Urology, Singapore General Hospital, Singapore, Singapore; 6 Department of Surgical and Perioperative Science, Urology and Andrology, Umeå University, Umeå, Sweden; 7 Department of Surgical Sciences, Uppsala University, Uppsala, Sweden

**Keywords:** Prostate cancer, Prognostic prediction, Cancer-specific mortality, Cambridge Prognostic Groups, Non-metastatic disease, Stratification, All-cause mortality, Competing risks, Improved treatment section, Treatment selection

## Abstract

**Background:**

The purpose of this study is to validate a new five-tiered prognostic classification system to better discriminate cancer-specific mortality in men diagnosed with primary non-metastatic prostate cancer.

**Methods:**

We applied a recently described five-strata model, the Cambridge Prognostic Groups (CPGs 1-5), in two international cohorts and tested prognostic performance against the current standard three-strata classification of low-, intermediate- or high-risk disease. Diagnostic clinico-pathological data for men obtained from the Prostate Cancer data Base Sweden (PCBaSe) and the Singapore Health Study were used. The main outcome measure was prostate cancer mortality (PCM) stratified by age group and treatment modality.

**Results:**

The PCBaSe cohort included 72,337 men, of whom 7162 died of prostate cancer. The CPG model successfully classified men with different risks of PCM with competing risk regression confirming significant intergroup distinction (*p* < 0.0001). The CPGs were significantly better at stratified prediction of PCM compared to the current three-tiered system (concordance index (C-index) 0.81 vs. 0.77, *p* < 0.0001). This superiority was maintained for every age group division (*p* < 0.0001). Also in the ethnically different Singapore cohort of 2550 men with 142 prostate cancer deaths, the CPG model outperformed the three strata categories (C-index 0.79 vs. 0.76, *p* < 0.0001). The model also retained superior prognostic discrimination in the treatment sub-groups: radical prostatectomy (*n* = 20,586), C-index 0.77 vs. 074; radiotherapy (*n* = 11,872), C-index 0.73 vs. 0.69; and conservative management (*n* = 14,950), C-index 0.74 vs. 0.73. The CPG groups that sub-divided the old intermediate-risk (CPG2 vs. CPG3) and high-risk categories (CPG4 vs. CPG5) significantly discriminated PCM outcomes after radical therapy or conservative management (*p* < 0.0001).

**Conclusions:**

This validation study of nearly 75,000 men confirms that the CPG five-tiered prognostic model has superior discrimination compared to the three-tiered model in predicting prostate cancer death across different age and treatment groups. Crucially, it identifies distinct sub-groups of men within the old intermediate-risk and high-risk criteria who have very different prognostic outcomes. We therefore propose adoption of the CPG model as a simple-to-use but more accurate prognostic stratification tool to help guide management for men with newly diagnosed prostate cancer.

**Electronic supplementary material:**

The online version of this article (10.1186/s12916-018-1019-5) contains supplementary material, which is available to authorized users.

## Background

Prostate cancer is a growing burden on health care systems worldwide [1, 2]. With rising disease awareness, an increasing proportion of men are presenting with non-metastatic disease [3, 4]. There is an urgent need to improve the prognostic precision for men with non-metastatic disease since management options are becoming more diversified, e.g. the increasing use of active surveillance for low-risk disease, and, conversely, due to the recognition that more intensive, combined treatment is needed in high-risk disease [5, 6]. Current risk stratification models were primarily developed to predict therapy failure and not the risk of prostate cancer death. Moreover, they are almost exclusively based on surgically and radiotherapy-treated men and do not include men who are managed conservatively [7, 8]. Nevertheless, the simple clinico-pathological variables that go into these models make them easy to use, and they are commonly the first triaging step recommended by many national and international guidelines for clinical decision-making [9–12].

To address this, we recently remodelled the components (histological grade, clinical stage and prostate-specific antigen (PSA) at diagnosis) that comprise the currently used risk classification systems [13]. In a new five-strata model, we also incorporated the new histological grade grouping recently recommended by the International Society of Urological Pathology (ISUP), which has been shown to be a better predictor of disease recurrence and progression than the Gleason sum alone [14, 15]. In a cohort of nearly 12,000 UK men, we found that the new model stratified the risk of prostate cancer death significantly better than the widely adopted three-tiered classification of low, intermediate and high risk [10–13]. In this paper, we report validation of this model, called the Cambridge Prognostic Groups (CPGs), in two separate, ethnically different cohorts: 72,337 Swedish men and 2550 men from a Southeast Asian population. Using the Swedish study group, we also assessed the utility of the CPG model in pre-treatment prognosis in men who had surgery, radiotherapy or conservative management.

## Methods

### Study cohorts

#### Prostate Cancer data Base Sweden

The Prostate Cancer data Base Sweden (PCBaSe) 3.0 was created through record linkages between the National Prostate Cancer Register (NPCR) of Sweden and several other population-based, nationwide health care registers and demographic databases [16]. The capture rate of the NPCR is 98% compared to that of the Swedish Cancer Registry, to which registration is mandated by law [17]. Information on the underlying and contributing causes and on the date of death was obtained from the Cause of Death Register, which captures all deaths in Sweden. The overall agreement between the Cause of Death Register and reviewed medical records is approximately 86% (95% confidence interval 85–87%) [18]. PCBaSe does not include information on sub-categories for local clinical stages T2 and T3. We obtained data of 80,803 men in PCBaSe, accrued from 2000 to 2010, with no evidence of metastatic disease (Mx or M0) and PSA < 100 ng/ml. All men were followed until death, emigration or to 31 December 2015, whichever occurred first. The outcome event for each man was one of the following: alive, prostate cancer-specific death or other causes of death. To assess prognostic performance, each man was assigned to the appropriate CPG using diagnostic clinical parameters including PSA at diagnosis (nanograms per millilitre), clinical T stage and Gleason Grade Group as previously described [13] (Table 1). Because these criteria were essential to assigning a CPG category, 8466 men had to be excluded because of a lack of data in one or more of these fields. As a comparator we also assigned each individual to the appropriate group in the widely adopted three-strata model (low, intermediate or high risk) published in many national and international guidelines including the UK [10–12]. For this paper we have used the UK National Institute for Health and Care Excellence (NICE) version as the reference, which is itself based on the D’Amico criteria first published in 1997 [7, 10]. For treatment-specific analysis we focused on men managed by primary radical prostatectomy, radical radiotherapy or conservative management. We did not have available data on any subsequent treatments men may have received or the use of concurrent adjuvant treatments. In the PCBaSe cohort, the term conservative management was until 2008 used to denote both active surveillance and watchful waiting. For simplicity, we have therefore also used the term conservative management for treatments that from 2008 were categorised as either active surveillance or watchful waiting. Any individuals with missing data fields were excluded from the analysis, as all components were crucial to assigning a prognostic group. Median follow-up was 7 years, and 51% of the cohort had follow-up for 10 years or until death. Ethical permission for data collection for PCBaSe was provided by the Research Ethics Board at Umeå University.

**Table 1:** Criteria of the new Cambridge Prognostic Groups for non-metastatic prostate cancer

Cambridge Prognostic Group (CPG)	Criteria
1	Gleason score 6 (Grade Group 1) *AND* PSA < 10 ng/ml *AND* Stages T1–T2
2	Gleason score 3 + 4 = 7 (Grade Group 2) *OR* PSA 10–20 ng/ml *AND* Stages T1–T2
3	Gleason score 3 + 4 = 7 (Grade Group 2) *AND* PSA 10–20 ng/ml *AND* Stages T1–T2 *OR* Gleason 4 + 3 = 7 (Grade Group 3) *AND* Stages T1–T2
4	One of Gleason score 8 (Grade Group 4) *OR* PSA > 20 ng/ml *OR* Stage T3
5	Any combination of Gleason score 8 (Grade Group 4), PSA > 20 ng/ml or Stage T3 *OR* Gleason score 9–10 (Grade Group 5) *OR* Stage T4

#### Singapore General Hospital prostate cancer database

The Singapore Health Study identified incident prostate cancer cases and deaths amongst cohort members by record linkage of the cohort database with the population-based Singapore Cancer Registry and the Singapore Registry of Births and Deaths. Dedicated cancer registrars prospectively collected and maintained data on all prostate cancers diagnosed and/or treated at Singapore General Hospital. Ethics for data collection and use is covered by CIRB ref. 2009/1053/D approved by the Singhealth Centralised Institutional Review Board. As in the PCBaSe cohort, only men with no evidence of metastatic disease and PSA < 100 ng/ml were included and stratified by the CPG or three-strata model (final cohort size of 2550). Median follow-up was shorter at 4.1 years, and 21.1% of the cohort had follow-up for 10 years or until death. As before, each patient’s case outcome was recorded as alive or prostate cancer-specific death or other causes of death. All data was anonymised at the source at both international centres before being used for analysis.

### Statistical analysis

The statistical methodology closely followed our first publication on the model, and the setting, eligibility criteria, outcome and predictors were similar to those of our development cohort [13]. The primary outcome of interest was the risk of prostate cancer mortality (PCM). All-cause mortality was also recorded. To study survival differences between prognostic groups, we applied a Cox proportional hazards regression model and the log rank test with pair-wise comparisons. “Low risk” was the reference group in the NICE model and “CPG1” in the CPGroup model. The null hypothesis was no difference between groups in the probability of prostate cancer death. For visual comparison and to explore estimation of survival time, cumulative incidence curves were constructed. Competing hazards risk regression using the Fine-Gray test was applied to include the influence of non-cancer deaths on model performance. For model discrimination, we used the somersd package to compute the rank parameters concordance index. Sub-hazard ratios were used in computation instead of hazard ratio to account for competing risks from other causes of death [19]. We then compared the performance of the new model to the three-tiered NICE groups. In the PCBaSe cohort, we further explored prognostic performance by stratifying patients according to three age groups (< 60, 60–69 and ≥ 70 years old). We also investigated the CPG model’s prognostic performance by treatment types, focusing on radical prostatectomy, radical radiotherapy and conservative management. For each modality, we computed hazard ratios and concordance index (C-index) as before with inclusion of competing risks of death similarly as in the initial study [13]. Statistical analyses were performed using *Stata Statistical Software, Release 14* (StataCorp LP, 2015, College Station, TX, USA).

## Results

### Prognostic performance in the PCBaSe cohort

**Fig. 1:**
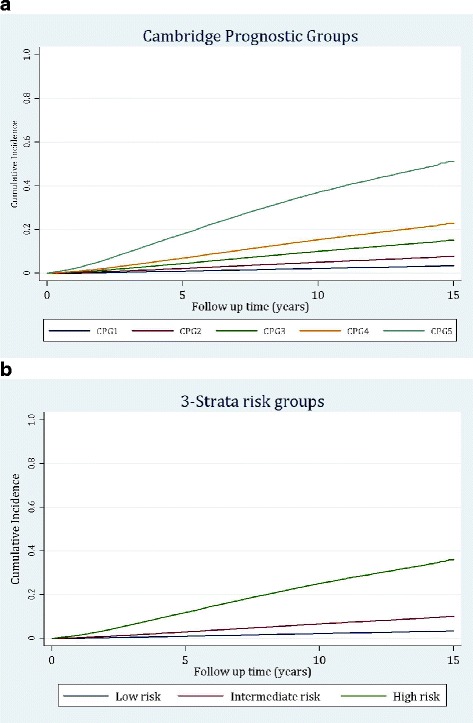
Cumulative incidence curves for prostate cancer-specific survival in the PCBaSe cohort (*n* = 72,337) stratified by the **a** Cambridge Prognostic Groups and **b** current three-strata risk groups as a comparator model

**Fig. 2:**
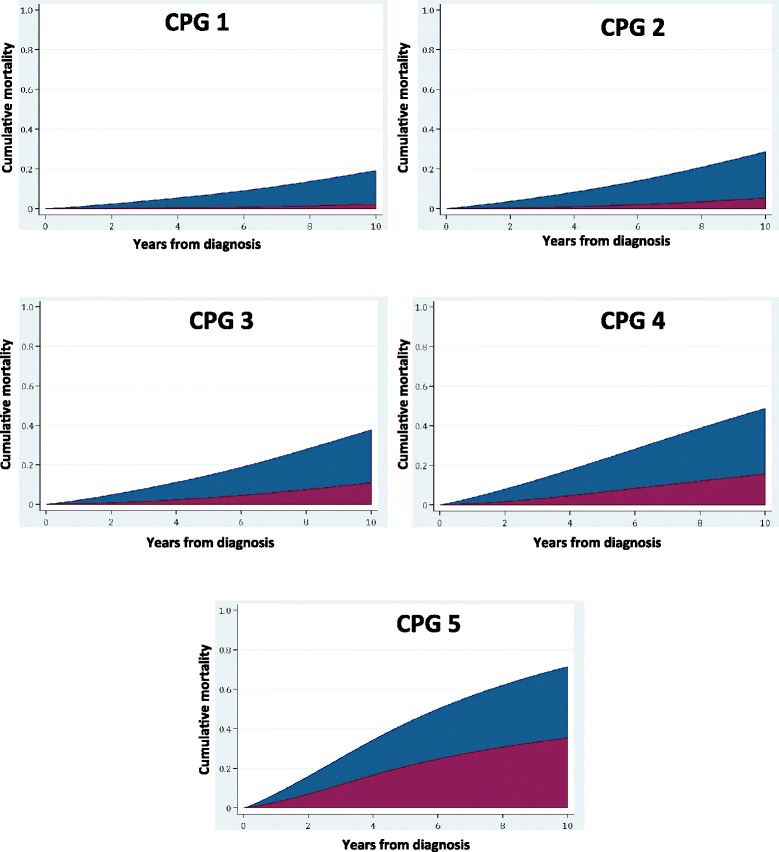
Ten-year prostate cancer and other-cause mortality rates stratified by each Cambridge Prognostic Group (CPG) category in the PCBaSe cohort (*n* = 72,337). ***Red*** prostate cancer mortality, ***blue*** other-cause mortality

The final PCBaSe cohort included 72,337 men, of whom 7162 died of prostate cancer. A further 15,921 men died of other causes during follow-up. Forty-five percent received radical treatment, either by radical prostatectomy (*n* = 20,586) or radical radiotherapy (*n* = 11,872) while 14,950 (21%) were managed conservatively. The remainder had other treatments or had missing records (Additional file 1: Table S1). Additional file 2: Table S2 shows the distribution of the Swedish cohort by age and diagnostic clinico-pathological variables. In this cohort, the CPG model classified men into the five sub-groups with very different risks of PCM in a competing risk analysis (*p* < 0.0001 for all groups) (Table 2). Pair-wise competing risk regression also confirmed significant intergroup discrimination (*p* < 0.0001 for all comparisons) (Table 3). Visual assessment of model discrimination is further shown by the cumulative incidence curves in Fig. 1a demonstrating clear differences in outcomes between the groups. The corresponding curves for the standard three-strata model are shown in Fig. 1b. These results reproduce our initial findings and confirm the utility of the CPG sub-groups as a valuable prognostic tool at the time of prostate cancer diagnosis [13]. The cumulative comparative 10-year mortality rates from prostate cancer and other causes of death are shown for each CPG category in Fig. 2. We tested how the overall prognostic performance of the CPG model compared to the NICE categories. In the PCBaSe cohort, the NICE groups demonstrated a C-index from competing risk analysis of 0.77 for predicting PCM. The CPG model, however, had a significantly superior C-index of 0.81 (*p* < 0.001) (Table 4). Finally, we tested if model performance was influenced by patient age. In this analysis the CPG model again outperformed the NICE model in every age group tested (Additional file 3: Table S3).

**Table 2:** Distribution of cases/deaths and sub-hazard ratios from competing risk analysis for each Cambridge Prognostic Group (CPG) category in the PCBaSe cohort (*n* = 72,337)

CPG	Number of men	Deaths from prostate cancer	Deaths from other causes	Sub-hazard ratio (95% confidence interval)	*p* value
1	25,303	482	3740	Reference	NA
2	14,796	628	2912	2.30 (2.04–2.59)	< 0.0001
3	7354	589	1532	4.70 (4.17–5.30)	< 0.0001
4	13,506	1831	4011	7.42 (6.71–8.19)	< 0.0001
5	11,378	3632	3726	20.52 (18.66–22.55)	< 0.0001
Total	72,337	7162	15,921	_	_

**Table 3:** Competing risk regression analysis of Cambridge Prognostic Groups (CPGs) in the PCBaSe cohort

CPG comparison	Sub-hazard ratio	95% confidence interval	*p* value
2 vs. 1	2.30	2.04–2.59	< 0.0001
3 vs. 2	2.11	1.89–2.36	< 0.0001
4 vs. 3	1.56	1.42–1.72	< 0.0001
5 vs. 4	2.72	2.58–2.88	< 0.0001

**Table 4:** Concordance indices of the current three-strata risk group model (NICE) and Cambridge Prognostic Group (CPG) from competing risk analysis in predicting prostate cancer-specific mortality (*p* < 0.001 for both comparisons)

	Concordance index (confidence interval)	
Cohort (*n*)	NICE	CPG
PCBaSe (72,337)	0.77 (0.76–0.77)	0.81 (0.81–0.82)
Singapore (2550)	0.76 (0.73–0.80)	0.79 (0.76–0.83)

### Prognostic performance in a Singapore cohort

We next sourced a separate cohort of 2550 men from Singapore of very different ethnicity (Chinese *n* = 2137, Indian *n* = 136, Malay *n* = 143, others *n* = 134). In this cohort there were 142 prostate cancer deaths and 266 deaths from other causes. Men were predominantly treated by radical therapy (72%): radical prostatectomy (*n* = 1012), radical radiotherapy (*n* = 824). Another 539 (21%) were managed conservatively. Additional file 4: Table S4 shows the distribution by clinical pathological variables. In this cohort, the CPG model continued to show progressively higher hazard ratios except between CPG1 and CPG2, where there were only 12 prostate cancer deaths (Additional file 5: Table S5). Overall, the model prognostic performance from competing risk analysis was again significantly superior to that of the three-strata system, with a C-index of 0.79 vs. 0.76 (*p* < 0.001) (Table 4).

### Clinical utility of the CPG model in treatment-specific prognosis

Sub-analysis of the CPG performance across different treatment settings was performed in the PCBaSe cohort. Amongst the radical therapy groups, sub-dividing traditional intermediate-risk disease into CPG2 and CPG3 identified men with very different PCM outcomes (*p* < 0.0001, Additional file 6: Table S6 and Additional file 7: Table S7). Notably, CPG3 (two intermediate-risk factors or histological Grade Group 3 alone) conferred a much poorer outcome compared to CPG2 regardless of whether men had surgery or radiotherapy (Additional file 8: Table S8). Indeed, pair-wise competing risk regression showed that CPG3 PCM outcomes were generally very similar to that of CPG4 (men with a single high-risk factor). In both radical treatment groups, however, men in CPG5 had the worst outcomes. Even compared to CPG4 alone these men had a nearly threefold higher risk of PCM (Additional file 6: Table S6, Additional file 7: Table S7 and Additional file 8: Table S8). Amongst men who had conservative management, only 3% of men in CPG1 died of prostate cancer, reinforcing the overwhelming indolent behaviour of cancers in this sub-group. For intermediate-risk disease it was particularly notable that men with CPG3 had a more than twofold higher risk of prostate cancer death compared to men in CPG2. The number of men who had conservative management in CPGs 3–5 was, however, low (representing only 20% of this sub-cohort); hence, more detailed interpretation of these groups was deemed unreliable. Overall, the CPG model again consistently outperformed the current three-strata risk groups in terms of prognostic performance regardless of treatment type (Table 5). Additional file 9: Table S9 shows the comparative cancer-related mortality between the three treatment options categorised by CPG sub-groups. Additional file 10: Table S10 shows the cross tabulation of distribution between the three-strata model and the CPG criteria.

**Table 5:** Concordance indices of the current three-strata risk group model (NICE) and Cambridge Prognostic Group (CPG) from competing risk analysis in predicting prostate cancer-specific mortality stratified by each treatment group (*p* < 0.0001 for all comparisons)

Cohort (*n*)	Concordance index (confidence interval)	
	NICE	CPG
Prostatectomy (20,586)	0.74 (0.72–0.77)	0.77 (0.74–0.79)
Radiotherapy (11,872)	0.69 (0.67–0.70)	0.73 (0.71–0.75)
Conservative management (11,757)	0.73 (0.71–0.74)	0.74 (0.73–0.76)

## Discussion

The CPG model, now tested in three different international cohorts in two studies including 86,732 primary prostate cancers, delivers distinct cancer mortality sub-groups with a high prognostic accuracy. The prognostic power of the model was very consistent between our development cohort and this validation study [13]. To our knowledge, the CPG model is the first risk stratification tool to have been derived from and validated in cohorts of newly diagnosed men using cancer death as the primary outcome. Our tested cohorts also included significant proportions of locally advanced cases (12 and 16%) and men managed conservatively (19 and 21%), which reflect most real-world practices where PSA screening is uncommon and unlikely to be implemented [5, 20–22].

The intermediate-risk group is the largest category of patients in contemporary cohorts [5]. The CPG model divides this group into two categories: CPG2, which is associated with a relatively good prognosis, and CPG3 (a combination of intermediate-risk factors or Gleason Grade Group 3 on its own), with a substantially higher mortality risk despite radical therapy. This data supports the recent work of Raldow *et al.*, where men with multiple intermediate-risk features had higher rates of prostate cancer death following brachytherapy [23]. Our results further suggest that many men with CPG2 disease may potentially be candidates for conservative management, at least initially, and thus avoid the morbidity of unnecessary treatment. In contrast, men with CPG3 should not be managed conservatively, as they have a much higher baseline risk of PCM. We do interpret this with caution, as our data may be potentially biased by treatment selection. Nevertheless, our results are supported by the work of Musunura *et al.*, who observed a similar worse survival outcome from active surveillance in men with a combination of Gleason 7 and a high PSA [24]. Our distinction between CPG2 and CPG3 has now also been independently identified by the new 2017 American Urological Association (AUA)/American Society for Radiation Oncology (ASTRO)/Society of Urologic Oncology (SUO) localised prostate cancer guidelines. They have defined a favourable and unfavourable category amongst intermediate-risk cancers, the criteria of which perfectly match the ones used here in the CPG2 and CPG3 categories [25]. Although the AUA/ASTRO/SUO definitions were not derived from primary research, they endorse our evidence-based distinction from a large cohort study that these sub-groups are linked to very different mortality outcomes. Consistently across all treatments, the split of the traditional high-risk category into CPG4 and CPG5 (multiple high-risk features) defined groups with very different risks of cancer death. CPG5 men had more than double the risk of PCM, even when compared to CPG4. These results support the findings of previous studies reporting that multiple high-risk factors confer a much worse treatment-specific outcome [26, 27]. Our study is, however, the first to show this effect in a very large cohort and simultaneously across different treatment types. Men with CPG3 and CPG4 disease represented statistically different prognostic sub-groups in our overall cohort analysis with distinctly different outcomes in intergroup comparisons. This mirrors the findings of our initial development study [13]. However, we do show for the first time that they may have very similar outcomes when treated by radical therapy. The reason for this may be that these treatment sub-cohorts were too small to pick up a difference, but our findings do support the notion that CPG3 likely represents a distinct aggressive sub-type of intermediate-risk disease more akin to the traditional high-risk disease designation.

A consistent criticism of risk and prognostic groupings is that they do not address intra-group heterogeneity [28]. As an example, Joniau *et al.* showed that amongst very high-risk men (in our study, CPG5) having surgery, the sub-group T3 and PSA > 20 had better outcomes compared to men with very high Gleason score 9–10 disease [27]. Although this criticism could also be applied to the CPG, we believe that our stratification system is an important first step in providing a more accurate but still simple framework for more individualised decision-making in non-metastatic prostate cancer. Hence, when we looked at our very high-risk group, the different categories did all have significantly worse mortality outcomes compared to CPG4 (the next prognostic level), *p* < 0.001 in all comparisons. In terms of practical usage, we believe that the CPG groups add significant clinical benefit. For example, men in CPG1 should be preferentially steered towards active surveillance. Many men in CPG2 are also likely to do well from this option but may need a more intensive surveillance schedule. In contrast, men in CPG3 and CPG4 clearly need curative therapy, and for these men the added use of individualised estimates of treatment outcomes could be very helpful. Bespoke biomarkers could also be used which are more appropriate for the disease context. A recent example is the work of Ahmad *et al.,* who showed that adding a DNA methylation index to the Cancer of the Prostate Risk Assessment (CAPRA) score improved prediction of PCM in men with intermediate-risk disease (area under the curve (AUC) from 0.62 to 0.74) [29]. Fraser *et al.* also studied men with intermediate-risk disease having radical therapy and demonstrated the utility of a panel of 40 recurrent genomic alterations in identifying those at highest risk of treatment failure [30]. Hence in the future, improved outcome discrimination within the CPG sub-groups might be gained by including such factors to add granularity. Men in CPG5 in particular clearly need a more aggressive and new approach to treatment and may be the ideal cohort for molecular sub-typing and targeted neo-adjuvant drugs combined with radical therapy when planning new clinical trials [31]. Conversely, it is likely to be a waste of resources to do such profiling in men with already good outcomes (e.g. those in CPG1). The CPG model may also be used to construct tailored follow-up protocols. For instance, men with CPG5 disease are likely to benefit most from early adjuvant treatment after radical therapy compared to men with CPG4 because of a much higher risk of a poor outcome. Conversely, in a surveillance programme, men in CPG1 are likely to only need a low-intensity follow-up schedule. A trigger for conversion to treatment might then be an increment to a higher CPG category during follow-up evaluation.

Our study does have limitations. It has been built and validated on men who have been diagnosed via trans-rectal ultrasound-guided biopsy, which is known to underestimate true histological grade and overall tumour burden [32]. However, the contribution that more intensive biopsy schema might make is currently uncertain. The ProtecT Study, for instance, showed extremely low mortality rates at 10 years in the surveillance cohort, despite the fact that men only had this kind of biopsy and at least a third likely harboured missed higher risk disease [33]. We did not have data on biopsy core involvement in our cohorts, and it was not a requirement for our model; thus, we cannot say if such granularity would improve its prognostic power. We note that biopsy core involvement is not included in contemporary guidelines outside the USA, and there is no international consensus on its use [10–12]. We also did not sub-classify within T stages, but we have previously noted the inaccuracies in its standard clinical use [34]. Our cohort predates the use of magnetic resonance imaging (MRI) for guided biopsies, which is already changing clinical practice [35, 36]. The CPG model, however, will retain utility regardless of the biopsy approach, as it is based on standard clinico-pathological variables. Indeed, we have already demonstrated the use of the model with MRI-based staging in predicting bone metastasis at diagnosis [37]. About 11% of the PCBaSe cohort had to be excluded, as we did not have all the clinico-pathological details. Details of how missing data is handled in PCBaSe have been previously reported [38]. Finally, although we have included competing mortality risks, our model does not include co-morbidity as a variable. Indeed, none of the current UK and European prostate cancer guidelines do so [10–12]. The US National Comprehensive Cancer Network (NCCN) guidelines also only go as far as to distinguish a life expectancy of less or more than 5 years [39]. The CPG model can of course be used alongside other tools to predict other-cause mortality [40].

## Conclusions

In summary, here we have confirmed the superiority of a five-tiered prognostic system over the prevailing three-tiered model in better stratifying prognosis in men with non-metastatic prostate cancer. Our model is unique in that it has been built from primary diagnostic source data linked to PCM outcomes, it has been tested across different treatment types and now has been validated in very large cohorts of men. It is notable that in the USA the NCCN and new AUA/ASTRO/SUO localised prostate cancer guidelines are both also endorsing five-strata systems for non-metastatic disease although with different sub-categories [25, 38]. While the AUA/ASTRO/SUO endorse splitting the old intermediate-risk category, they do not recommend a very high-risk sub-category. The NCCN criteria, in contrast, endorse a very high-risk category but do not endorse splitting the intermediate-risk group. The CPG model underscores the clinical relevance of sub-stratification of the old intermediate-risk group in the new AUA/ASTRO/SUO guidelines as well as the designation of a very high-risk category but combines these two new sub-groups in the only single, easy-to-reference, evidence-based model. While the prognostic performance of the CPG model has remained consistent in three cohorts from different countries, future independent validation would strengthen its clinical utility. Our model, however, can be used by any clinician anywhere in the world without requiring any additional data or costs. Future work will determine how additional variables, including biopsy data and molecular profiles, can be added to further individualise prognostic prediction both across and within treatment sub-types.

## Additional files

Additional file 1: **Table S1.** Use of treatments according to Cambridge Prognostic Group (CPG) in the PCBaSe cohort. (DOCX 15 kb)

Additional file 2: **Table S2.** Distribution of the PCBaSe study cohort (*n* = 72,337) by age, serum PSA at presentation, biopsy Grade Group (GG) and clinical stage (PSA in ng/ml). (DOCX 16 kb)

Additional file 3: **Table S3.** Concordance indices of the current three-strata risk group model (NICE) and Cambridge Prognostic Group (CPG) from competing risk analysis in predicting prostate cancer-specific mortality stratified by age groups in the PCBaSe cohort (*p* < 0.001 for all comparisons). (DOCX 14 kb)

Additional file 4: **Table S4.** Distribution of the Singapore study cohort (*n* = 2550) by age, PSA at presentation, biopsy Grade Group (GG) and clinical stage (PSA in ng/ml). (DOCX 15 kb)

Additional file 5: **Table S5.** Distribution of cases/deaths and sub-hazard ratios from competing risk analysis for each Cambridge Prognostic Group (CPG) in the Singapore cohort (*n* = 2550). (DOCX 15 kb)

Additional file 6: **Table S6.** Distribution of cases and deaths from prostate cancer and hazard ratios for each Cambridge Prognostic Group (CPG) in the PCBaSe radical prostatectomy cohort (*n* = 20,586). (DOCX 15 kb)

Additional file 7: **Table S7.** Distribution of cases and deaths from prostate cancer and hazard ratios for each Cambridge Prognostic Group (CPG) in the PCBaSe radical radiotherapy cohort (*n* = 11,872). (DOCX 15 kb)

Additional file 8: **Table S8.** Competing risk regression analysis of the Cambridge Prognostic Group (CPG) by treatment type. **A**. Radical prostatectomy cohort (*n* = 20,586), **B.** radical radiotherapy cohort (*n* = 11,872) and **C.** conservative management cohort (*n* = 14,950). Intergroup comparisons are shown. (DOCX 17 kb)

Additional file 9: **Table S9.** Comparative 10-year prostate cancer mortality rate per 1000 men stratified by treatment type and CPG category in the PCBaSe cohort (*n* = 72,337) (DOCX 15 kb)

Additional file 10: **Table S10.** Cross tabulation of the CPG and three-strata NICE criteria to show the sub-distributions of the cases between the two models in the PCBaSe cohort (*n* = 72,337). (DOCX 15 kb)

## Abbreviations

AUA/ASTRO/SUO: American Urological Association/American Society for Radiation Oncology/Society of Urologic Oncology
AUC: Area under the curve
C-index: Concordance index
CPG: Cambridge Prognostic Group
GG: Grade Group
MRI: Magnetic resonance imaging
NCCN: National Comprehensive Cancer Network
NICE: National Insititue for Clinical Excellence
PCBaSe: Prostate Cancer data Base Sweden
PCM: Prostate cancer mortality
PSA: Prostate-specific antigen
