# Supplementary material for: The Cambridge Prognostic Groups for improved prediction of disease mortality at diagnosis in primary non-metastatic prostate cancer: a validation study
Source: BMC Med. 2018 Feb 28;16:31. doi: 10.1186/s12916-018-1019-5 (PMC5831573; doi:10.1186/s12916-018-1019-5)
Supplement: Supplementary file 1 — Table S1. Use of treatments according to Cambridge Prognostic Group (CPG) in the PCBaSe cohort. (DOCX 15 kb) [file 12916_2018_1019_MOESM1_ESM.docx]

**Supplementary Table S1** – Use of treatments according to Cambridge Prognostic Group (CPG) in the PCBaSe cohort.

| **Treatment type** | **CPG 1** | **CPG 2** | **CPG 3** | **CPG 4** | **CPG 5** |
| --- | --- | --- | --- | --- | --- |
| **Radical Prostatectomy**  **Radical Radiotherapy**  **Conservative management**  **Androgen Deprivation***  **Other treatments**  **Not available/recorded** | 9890  2823  10799  769  392  630 | 5622  2494  4677  1354  238  411 | 2529  1579  1477  1415  107  247 | 2008  3096  2814  4933  224  431 | 537  1880  932  7624  131  274 |

*Given as primary and only therapy
