# Supplementary material for: The Cambridge Prognostic Groups for improved prediction of disease mortality at diagnosis in primary non-metastatic prostate cancer: a validation study
Source: BMC Med. 2018 Feb 28;16:31. doi: 10.1186/s12916-018-1019-5 (PMC5831573; doi:10.1186/s12916-018-1019-5)
Supplement: Supplementary file 2 — Table S2. Distribution of the PCBaSe study cohort (n = 72,337) by age, serum PSA at presentation, biopsy Grade Group (GG) and clinical stage (PSA in ng/ml). (DOCX 16 kb) [file 12916_2018_1019_MOESM2_ESM.docx]

**Supplementary Table S2** – Distribution of the PCBaSe study cohort (n=72,337) by age, serum PSA at presentation, biopsy Grade Group (GG) and clinical stage. (PSA in ng/ml)

|  |  |  |  |  | |  |  |  |
| --- | --- | --- | --- | --- | --- | --- | --- | --- |
| **Age (y)** | **n** | **PSA** | **n** | **Biopsy grade** | | **n** | **Stage** | **N** |
|  |  |  |  |  |  |  |  |  |
| **<60** | 10309 | **<10** | 38,690 | **<6** | GG 1 | 39,572 | **T1** | 37270 |
| **60-69** | 28903 | **10-20** | 18,357 | **3+4** | GG 2 | 14,112 | **T2** | 23,473 |
| **70-79** | 23483 | **>20** | 15,290 | **4+3** | GG 3 | 7892 | **T3** | 10,825 |
| **≥ 80** | 9642 |  |  | **8** | GG 4 | 6527 | **T4** | 769 |
|  |  |  |  | **9-10** | GG 5 | 4234 |  |  |
