# Supplementary material for: The Cambridge Prognostic Groups for improved prediction of disease mortality at diagnosis in primary non-metastatic prostate cancer: a validation study
Source: BMC Med. 2018 Feb 28;16:31. doi: 10.1186/s12916-018-1019-5 (PMC5831573; doi:10.1186/s12916-018-1019-5)
Supplement: Supplementary file 3 — Table S3. Concordance indices of the current three-strata risk group model (NICE) and Cambridge Prognostic Group (CPG) from competing risk analysis in predicting prostate cancer-specific mortality stratified by age groups in the PCBaSe cohort (p < 0.001 for all comparisons). (DOCX 14 kb) [file 12916_2018_1019_MOESM3_ESM.docx]

**Supplementary Table S3** – Concordance indices of the current 3 strata risk group model (NICE) and Cambridge Prognostic Group (CPG) from competing risk analysis in predicting prostate cancer specific mortality stratified by age groups in the PCBaSe cohort (p<0.001 for all comparisons).

|  | **Concordance index (confidence interval)** | |
| --- | --- | --- |
| **Cohort (n)** | **NICE** | **CPG** |
|  |  |  |
| Age <60 years (10,309) | 0.84 (0.82-0.86) | 0.87 (0.85-0.89) |
| Age 61-69 years (31,719)  Age > 70 years (30,309) | 0.78 (0.77-0.79)  0.69 (0.68-0.69) | 0.82 (0.81-0.83)  0.74 (0.73-0.75) |
|  |  |  |
