# Supplementary material for: The Cambridge Prognostic Groups for improved prediction of disease mortality at diagnosis in primary non-metastatic prostate cancer: a validation study
Source: BMC Med. 2018 Feb 28;16:31. doi: 10.1186/s12916-018-1019-5 (PMC5831573; doi:10.1186/s12916-018-1019-5)
Supplement: Supplementary file 4 — Table S4. Distribution of the Singapore study cohort (n = 2550) by age, PSA at presentation, biopsy Grade Group (GG) and clinical stage (PSA in ng/ml). (DOCX 15 kb) [file 12916_2018_1019_MOESM4_ESM.docx]

**Supplementary Table S4**– Distribution of the Singapore study cohort (n=2550) by age, PSA at presentation, biopsy Grade Group (GGG) and clinical stage (PSA in ng/ml)

|  |  |  |  |  | |  |  |  |
| --- | --- | --- | --- | --- | --- | --- | --- | --- |
| **Age (y)** | **N** | **PSA** | **n** | **Biopsy grade group** | | **N** | **Stage** | **N** |
|  |  |  |  |  |  |  |  |  |
| **<60** | 501 | **<10** | 1344 | **<6** | GG 1 | 1127 | **T1** | 1626 |
| **60-69** | 1198 | **10-20** | 682 | **3+4** | GG 2 | 723 | **T2** | 661 |
| **70-79** | 739 | **>20** | 524 | **4+3** | GG 3 | 327 | **T3** | 246 |
| **≥ 80** | 112 |  |  | **8** | GG 4 | 170 | **T4** | 17 |
|  |  |  |  | **9-10** | GG 5 | 203 |  |  |
