# Supplementary material for: The Cambridge Prognostic Groups for improved prediction of disease mortality at diagnosis in primary non-metastatic prostate cancer: a validation study
Source: BMC Med. 2018 Feb 28;16:31. doi: 10.1186/s12916-018-1019-5 (PMC5831573; doi:10.1186/s12916-018-1019-5)
Supplement: Supplementary file 5 — Table S5. Distribution of cases/deaths and sub-hazard ratios from competing risk analysis for each Cambridge Prognostic Group (CPG) in the Singapore cohort (n = 2550). (DOCX 15 kb) [file 12916_2018_1019_MOESM5_ESM.docx]

**Supplementary Table S5** – Distribution of cases/deaths and sub-hazard ratios from competing risk analysis for each Cambridge Prognostic Group (CPG) in the Singapore cohort (n= 2550).

|  |  |  |  |  |  |
| --- | --- | --- | --- | --- | --- |
| **CPG** | **Number of men** | **Deaths from prostate cancer** | **Deaths from other causes** | **Sub-Hazard Ratio (95% CI)** | **p value** |
| **1** | 734 | 5 | 58 | Ref | NA |
| **2** | 621 | 7 | 51 | 1.79 (0.57-5.64) | 0.32 |
| **3** | 386 | 12 | 25 | 4.89 (1.70-14.06) | 0.003 |
| **4** | 430 | 37 | 65 | 8.75 (3.42-22.37) | <0.0001 |
| **5** | 379 | 52 | 39 | 22.78 (9.12-56.90) | <0.0001 |
|  |  |  |  |  |  |
