# Supplementary material for: The Cambridge Prognostic Groups for improved prediction of disease mortality at diagnosis in primary non-metastatic prostate cancer: a validation study
Source: BMC Med. 2018 Feb 28;16:31. doi: 10.1186/s12916-018-1019-5 (PMC5831573; doi:10.1186/s12916-018-1019-5)
Supplement: Supplementary file 6 — Table S6. Distribution of cases and deaths from prostate cancer and hazard ratios for each Cambridge Prognostic Group (CPG) in the PCBaSe radical prostatectomy cohort (n = 20,586). (DOCX 15 kb) [file 12916_2018_1019_MOESM6_ESM.docx]

**Supplementary Table S6** - Distribution of cases and deaths from prostate cancer and hazard ratios for each Cambridge Prognostic Group (CPG) in the PCBaSe radical prostatectomy cohort (n= 20586).

|  |  |  |  |
| --- | --- | --- | --- |
| **CPG** | **Number of men (deaths from prostate cancer)** | **Hazard Ratio (95% CI)** | **p value** |
|  |  |  |  |
| **1** | 9890 (67) | Ref | NA |
| **2** | 5622 (69) | 2.05 (1.47-2.88) | <0.0001 |
| **3** | 2529 (79) | 5.99 (4.32-8.30) | <0.0001 |
| **4** | 2008 (102) | 8.52 (6.26-11.60) | <0.0001 |
| **5** | 537 (68) | 23.87 (17.02-33.46) | <0.0001 |
|  |  |  |  |
