# Supplementary material for: The Cambridge Prognostic Groups for improved prediction of disease mortality at diagnosis in primary non-metastatic prostate cancer: a validation study
Source: BMC Med. 2018 Feb 28;16:31. doi: 10.1186/s12916-018-1019-5 (PMC5831573; doi:10.1186/s12916-018-1019-5)
Supplement: Supplementary file 7 — Table S7. Distribution of cases and deaths from prostate cancer and hazard ratios for each Cambridge Prognostic Group (CPG) in the PCBaSe radical radiotherapy cohort (n = 11,872). (DOCX 15 kb) [file 12916_2018_1019_MOESM7_ESM.docx]

**Supplementary Table S7** - Distribution of cases and deaths from prostate cancer and hazard ratios for each Cambridge Prognostic Group (CPG) in the PCBase radical radiotherapy cohort (n= 11872).

|  |  |  |  |
| --- | --- | --- | --- |
| **CPG** | **Number of men (deaths from prostate cancer)** | **Hazard Ratio (95% CI)** | **p value** |
|  |  |  |  |
| **1** | 2823 (39) | Ref | NA |
| **2** | 2494 (88) | 2.86 (1.96-4.17) | <0.0001 |
| **3** | 1579 (88) | 5.30 (3.63-7.74) | <0.0001 |
| **4** | 3096 (220) | 5.60 (3.98-7.88) | <0.0001 |
| **5** | 1880 (282) | 14.04 (10.04-19.63) | <0.0001 |
|  |  |  |  |
