# Supplementary material for: The Cambridge Prognostic Groups for improved prediction of disease mortality at diagnosis in primary non-metastatic prostate cancer: a validation study
Source: BMC Med. 2018 Feb 28;16:31. doi: 10.1186/s12916-018-1019-5 (PMC5831573; doi:10.1186/s12916-018-1019-5)
Supplement: Supplementary file 8 — Table S8. Competing risk regression analysis of the Cambridge Prognostic Group (CPG) by treatment type. A. Radical prostatectomy cohort (n = 20,586), B. radical radiotherapy cohort (n = 11,872) and C. conservative management cohort (n = 14,950). Intergroup comparisons are shown. (DOCX 17 kb) [file 12916_2018_1019_MOESM8_ESM.docx]

**Supplementary Table S8: A** - Competing risk regression analysis of the Cambridge Prognostic Group (CPG) by treatment type A: Radical prostatectomy cohort (n=20586). **B:** Radical radiotherapy cohort (n=11872) and **C:** Conservative management cohort (n=14950). Intergroup comparisons are shown.

**A**

| **CPG comparison** | **Sub-Hazard ratio** | **95% Confidence Interval** | | **p-value** |
| --- | --- | --- | --- | --- |
| 2 vs 1 | 2.07 | 1.48-2.90 | <0.0001 | |
| 3 vs 2 | 2.88 | 2.09-3.98 | <0.0001 | |
| 4 vs 3 | 1.45 | 1.08-1.95 | 0.01 | |
| 5 vs 4 | 2.73 | 2.01-3.71 | <0.0001 | |

**B**

| **CPG comparison** | **Sub-Hazard ratio** | **95% Confidence Interval** | **p-value** |
| --- | --- | --- | --- |
| 2 vs 1 | 2.85 | 1.95-4.14 | <0.0001 |
| 3 vs 2 | 1.88 | 1.40-2.52 | <0.0001 |
| 4 vs 3 | 1.08 | 0.84-1.38 | 0.549 |
| 5 vs 4 | 2.41 | 2.02-2.87 | <0.0001 |

**C**

| **CPG comparison** | **Sub-Hazard ratio** | **95% Confidence Interval** | **p-value** |
| --- | --- | --- | --- |
| 2 vs 1 | 2.48 | 2.11-2.92 | <0.0001 |
| 3 vs 2 | 1.81 | 1.47-2.22 | <0.0001 |
| 4 vs 3 | 1.05 | 0.87-1.92 | 0.577 |
| 5 vs 4 | 2.34 | 2.01-2.73 | <0.0001 |
