# Supplementary material for: The Cambridge Prognostic Groups for improved prediction of disease mortality at diagnosis in primary non-metastatic prostate cancer: a validation study
Source: BMC Med. 2018 Feb 28;16:31. doi: 10.1186/s12916-018-1019-5 (PMC5831573; doi:10.1186/s12916-018-1019-5)
Supplement: Supplementary file 9 — Table S9. Comparative 10-year prostate cancer mortality rate per 1000 men stratified by treatment type and CPG category in the PCBaSe cohort (n = 72,337) (DOCX 15 kb) [file 12916_2018_1019_MOESM9_ESM.docx]

**Supplementary Table S9** – Comparative 10 year Prostate Cancer Mortality rate per 1000 men stratified by treatment type and CPG category in the PCBase cohort (n=72,337)

|  | **CPG1** | **CPG2** | **CPG3** | **CPG4** | **CPG5** |
| --- | --- | --- | --- | --- | --- |
| **Treatment type** |  |  |  |  |  |
| Con Management | 7.06 | 19.75 | 30.82 | 37.07 | 75.19 |
| Radical Prostatectomy | 1.21 | 2.19 | 5.25 | 9.18 | 23.18 |
| Radical Radiotherapy | 2.10 | 6.13 | 10.16 | 14.25 | 28.94 |
