# Supplementary material for: The Cambridge Prognostic Groups for improved prediction of disease mortality at diagnosis in primary non-metastatic prostate cancer: a validation study
Source: BMC Med. 2018 Feb 28;16:31. doi: 10.1186/s12916-018-1019-5 (PMC5831573; doi:10.1186/s12916-018-1019-5)
Supplement: Supplementary file 10 — Table S10. Cross tabulation of the CPG and three-strata NICE criteria to show the sub-distributions of the cases between the two models in the PCBaSe cohort (n = 72,337). (DOCX 15 kb) [file 12916_2018_1019_MOESM10_ESM.docx]

**Supplementary Table S10** – Cross tabulation of the CPG and 3 strata NICE criteria to show the sub-distributions of the cases between the 2 models in the PCBase cohort (n= 72,337).

| **CPG** | **NICE** | | | **Total** |
| --- | --- | --- | --- | --- |
|  | **Low** | **Intermediate** | **High** |  |
| **1** | 25303 | 0 | 0 | 25303 |
| **2** | 0 | 14796 | 0 | 14796 |
| **3** | 0 | 7354 | 0 | 7354 |
| **4** | 0 | 0 | 13506 | 13506 |
| **5** | 0 | 0 | 11378 | 11378 |
| **Total** | 25304 | 22152 | 24887 | 72337 |
